# Stereolithography vs. Direct Light Processing for Rapid Manufacturing of Complete Denture Bases: An In Vitro Accuracy Analysis

**DOI:** 10.3390/jcm10051070

**Published:** 2021-03-04

**Authors:** Alexey Unkovskiy, Franziska Schmidt, Florian Beuer, Ping Li, Sebastian Spintzyk, Pablo Kraemer Fernandez

**Affiliations:** 1 Department of Prosthodontics, Geriatric Dentistry and Craniomandibular Disorders, Charité-Universitätsmedizin Berlin, Corporate Member of Freie Universität Berlin, Humboldt-Universität zu Berlin, Aßmannshauser Str. 4-6, 14197 Berlin, Germany; franziska.schmidt2@charite.de (F.S.); florian.beuer@charite.de (F.B.); 2 Department of Dental Surgery, Sechenov First Moscow State Medical University, Bolshaya Pirogovskaya Street, 19c1, 119146 Moscow, Russia; 3 Section Medical Materials Science and Technology, Tuebingen University Hospital, Osianderstr. 2-8, 72076 Tuebingen, Germany; ping.li@med.uni-tuebingen.de (P.L.); sebastian.spintzyk@med.uni-tuebingen.de (S.S.); 4 Department of Prosthodontics at the Centre of Dentistry, Oral Medicine and Maxillofacial Surgery with Dental School, Tuebingen University Hospital, Osianderstr. 2-8, 72076 Tübingen, Germany; pablo.kraemer-fernandez@med.uni-tuebingen.de

**Keywords:** stereolithography, direct light processing, complete denture, edentulism, rapid prototyping, 3D printing, additive manufacturing, rapid manufacturing

## Abstract

The topical literature lacks any comparison between stereolithography (SLA) and direct light processing (DLP) printing methods with regard to the accuracy of complete denture base fabrication, thereby utilizing materials certified for this purpose. In order to investigate this aspect, 15 denture bases were printed with SLA and DLP methods using three build angles: 0°, 45° and 90°. The dentures were digitalized using a laboratory scanner (D2000, 3Shape) and analyzed in analyzing software (Geomagic Control X, 3D systems). Differences between 3D datasets were measured using the root mean square (RMS) value for trueness and precision and mean and maximum deviations were obtained for each denture base. The data were statistically analyzed using two-way ANOVA and Tukey’s multiple comparison test. A heat map was generated to display the locations of the deviations within the intaglio surface. The overall tendency indicated that SLA denture bases had significantly higher trueness for most build angles compared to DLP (*p* < 0.001). The 90° build angle may provide the best trueness for both SLA and DLP. With regard to precision, statistically significant differences were found in the build angles only. Higher precision was revealed in the DLP angle of 0° in comparison to the 45° and 90° angles.

## 1. Introduction

With extensive applications of computer-aided design and computer-aided manufacturing (CAD/CAM) in modern clinical dentistry, various additive manufacturing (AM) methods have become available for the fabrication of surgical guides, dental models, provisional crowns and complete dentures [1,2,3]. In the past decade, a large number of clinical and technical protocols has been introduced to fabricate a complete denture in a fully digital workflow. Many of them utilize additive manufacturing for printing either a try-in or a definitive denture [4,5,6]. In a clinical study by Cristache et al., patients’ high levels of satisfaction with digitally produced dentures were recorded in a follow-up after 18 months [7]. The fabrication of complete dentures using AM can be considered as a promising technique with regard to its clinical and technical performance [8,9]. Furthermore, it was reported that additively produced denture bases show a comparable tissue adaptation to milled ones [10].

Direct light processing (DLP) is the most widely applied AM method for 3D printing of denture bases [4,10,11,12,13]. It utilizes a micro-mirror device and ultraviolet light for a layer-wise build-up of photopolymerizable resin [14]. Stereolithography (SLA) is an alternative vat polymerization method based on a laser beam raster scanning of the surface within a tank with photosensitive liquid, generally also a photopolymerizable resin [15,16].

Different principles in the printing process between SLA and DLP methods may potentially cause anisotropy with regard to the dimensional accuracy of the printed parts [17]. In case of SLA, the laser beam travels across the layer surface, causing localized polymerization of the photosensitive resin in the area of the illuminated field, whereas in case of DLP, the whole material portion in the x/y space is cured simultaneously by a one-time projection of the whole layer through the light projector [14]. Besides the type of resin and the light intensity, the accuracy of the mentioned AM methods may also be influenced by the build angle of the printing process [18,19,20]. This aspect has been investigated for DLP with regard to complete denture manufacturing and yielded no significant differences among the various build angles [12]. Another study highlighted potential differences in accuracy between DLP and SLA in the maxillofacial field [17]. Choi et al. reported that SLA may produce more accurate dental models than DLP [21]. The influence of build angle on the accuracy of SLA-produced surgical guides has also been widely investigated, reporting the 90° angle to yield the best clinical outcome [22]. Moreover, the layer thickness may influence the final result, whereby 50 µm layer printing provides a better dimensional accuracy [23].

However, no clinical report can be found regarding the utilization of SLA for manufacturing complete dentures in a digital workflow. The study of Hada et al. investigated the influence of the build angle on the accuracy of stereolithographically printed bases [24]. It must be emphasized that the material used in this study was transparent and not specified by the manufacturer for denture base fabrication.

However, there are various studies devoted to accuracy investigations of various SLA- and DLP-printed objects. However, the authors are unaware of any comparison between these AM methods with regard to their dimensional accuracy for a direct denture base fabrication in a fully digital workflow from certified denture base materials. Recently, You et al. compared SLA and DLP methods with regard to denture metal base fabrication, though within a semi-analog production chain [25].

Thus, the aim of the present study was to find out which of these vat polymerization techniques may produce the most accurate denture base for a fully digital workflow, in both cases using a certified denture base material and a uniform layer thickness. The study should also provide a recommendation for the printing preferences with regard to the build angle for this new application of SLA denture material. The potential finding of the study should aid a better understanding of how printing process parameters may influence the clinical performance of additively manufactured denture bases.

The first null hypothesis is that there will be no significant dimensional differences in SLA- and DLP-printed bases. Furthermore, it is hypothesized that the build angle of the SLA printing processes using the certified denture base material will not show any significant influence on the final dimensional accuracy.

## 2. Experimental Section

### 2.1. Specimen Design and Fabrication

**Figure 1:**
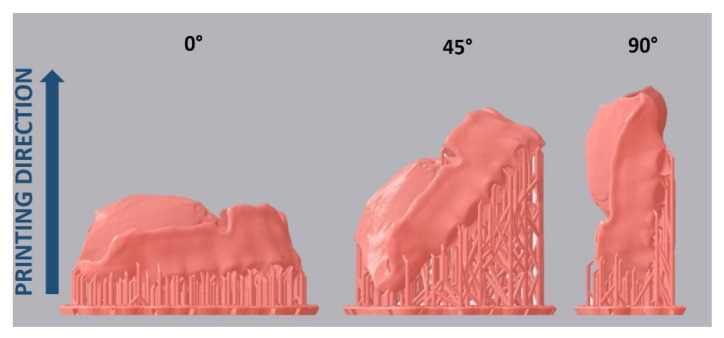
Nesting with three alternative build angles used in the study.

A maxillary complete denture was designed in CAD software (DentalCAD 2.3 Matera, exocad GmbH, Darmstadt, Germany) and exported in a surface tessellation language (STL) format. For the SLA group, the denture base data was imported into slicing software (PreForm, Formlabs, Somerville, MA, USA) and nested on the build platform in 0°, 45°, and 90° orientations (Figure 1).

**Figure 2:**
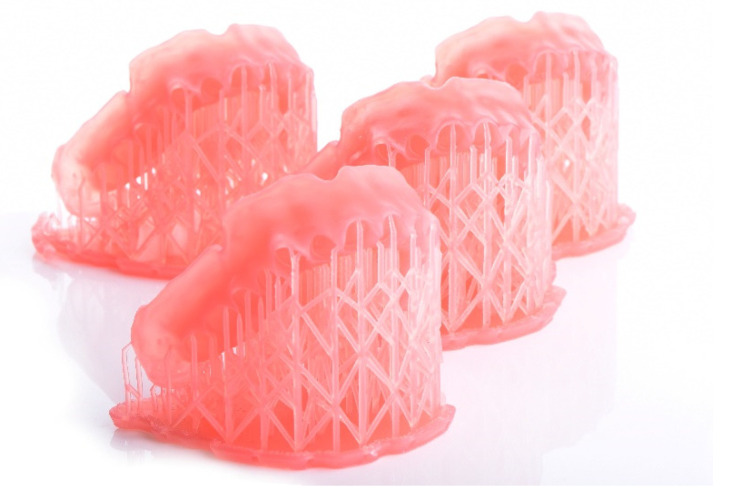
Exemplary 3D printed denture bases (here: stereolithography (SLA) with 45° build angle).

The supporting structures were generated automatically using the software script and then it was checked that none of them were connected to the intaglio surface. The denture bases were printed, *n* = 5 for each printing direction, with a liquid resin (Denture Base OP Resin, Formlabs, Somerville, USA) using an SLA printer (Form 3B, Formlabs, Somerville, MA, USA) (Figure 2). Afterwards, the bases were washed in isopropanol in a specific machine with the help of a stirrer to circulate the liquid (FormWash, Formlabs, Somerville, MA, USA) and photopolymerized in natural glycerin preheated to 80 °C for 60 min in a light chamber (FormCure, Formlabs, Somerville, MA, USA) according to the manufacturer’s specifications.

For the DLP group, the STL file was imported into slicing software (Netfabb Premium 2021, Autodesk, San Rafael, CA, USA). The supporting structures were also generated automatically using a software script for DLP printers and a base grid. Care was taken that none of them touched the intaglio surface. The denture bases were printed, *n* = 5 for each printing direction, with liquid resin (V-Print dentbase, VOCO GmbH, Cuxhaven, Germany) using a DLP printer (Solflex 350 PLUS, W2P Engineering GmbH, Vienna, Austria) with a flexible silicone vat (FlexVat, W2P Engineering GmbH, Vienna, Austria). Afterwards, the support structures were removed; the bases were washed out for 5 min in total in an ultrasonic cleaner with isopropanol, dried for 15 min and photopolymerized for 30 min in a light chamber (LC-3DPrint Box, 3D Systems Inc., Rock Hill, SC, USA) according to the manufacturer’s specifications.

### 2.2. Accuracy Analysis

**Figure 3:**
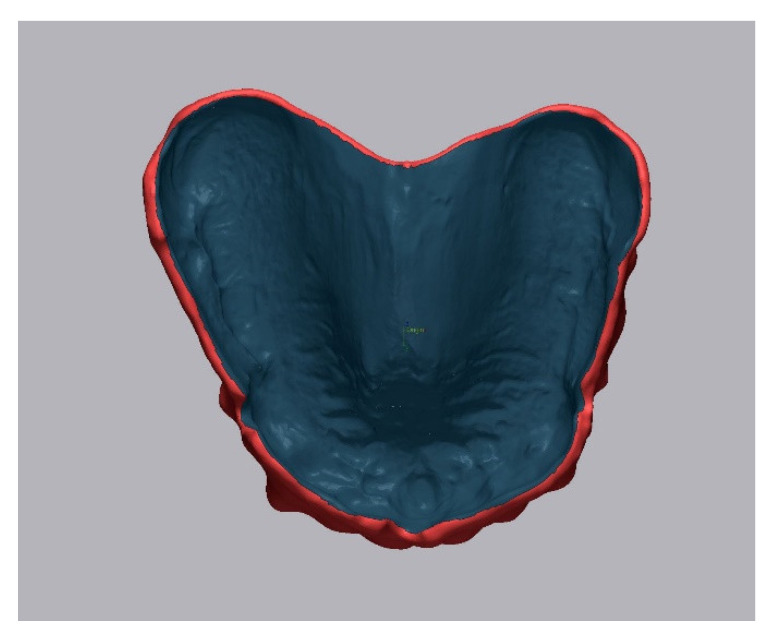
Segmentation of the reference dataset in Geomagic Control X (3D Systems) software for the further matching process. Only the intaglio surface (dark gray) was used for the best fit protocol.

The accuracy investigation encompassed trueness and precision analysis as per ISO 5725-1. The SLA- and DLP-printed denture bases were digitalized using a laboratory scanner (D2000, 3Shape, Copenhagen, Denmark). The gathered scans were exported in STL format and used for the accuracy test. For analysis of trueness, the obtained STL file of each printed denture was aligned with the reference CAD model using first the three-point-fit and then best-fit protocols in the analyzing software (Geomagic Control X, 3D Systems Inc., Rock Hill, SC, USA). For the alignment process, the intaglio surface was segmented from the remaining STL dataset, as shown in Figure 3. For the analysis of precision, the obtained STL files of each printed denture were matched to each other within each group.

For quantitative analysis of trueness and precision, the values were automatically calculated using the root mean square (RMS) error. RMS is recognized as a standard variable to measure differences between two 3D datasets [21]. The RMS deviation was calculated with the following formula:

(1) $$RMS\;=\;\sqrt{\frac{\sum_{i=1}^{n}{\left(x_{R,i}-x_{T,i}\right)}^{2}}{n}}$$

where *n* is the number of measured points, *x_R,i_* is the *i*-th measurement point of the reference model and *x_T,i_* is the measurement point of the dataset of the test model.

Furthermore, the mean and maximum deviations in mm were obtained for each dataset.

For qualitative analysis of trueness and precision, a heat map was generated for each dataset. The range of the maximum and minimum values was set to 1 mm. The tolerance level was set to ±0.025 mm as it represents the maximum z-axis resolution of the used AM methods of 0.05 mm.

For a better understanding of surface layering, the optical 3D metrology analysis with an optical scanner (Edge Master X, Alicona GmbH, Schönau am Königssee, Germany) of the palatal area of the intaglio surface was carried out. The scanning process was performed with 10× magnification lens under standard light conditions and was analyzed in 3D Image Viewer software (Alicona GmbH, Schönau am Königssee, Germany).

All gathered data were statistically analyzed in statistic software (JMP 14, SAS Corp., Heidelberg, Germany). First, the data were tested for normality by goodness of fit with the Shapiro–Wilk test. For normally distributed data, the statistical difference was analyzed by using two-way analysis of variance (ANOVA) with printing techniques and orientations as two independent factors. Tukey’s test was further performed for multiple comparison analysis. The threshold for significance was defined as a *p*-value less than 0.05.

## 3. Results

The mean differences between the RMS values, as well as mean and mean maximum deviation in mm for SLA and DLP denture bases, are shown in Table 1.

**Figure 4:**
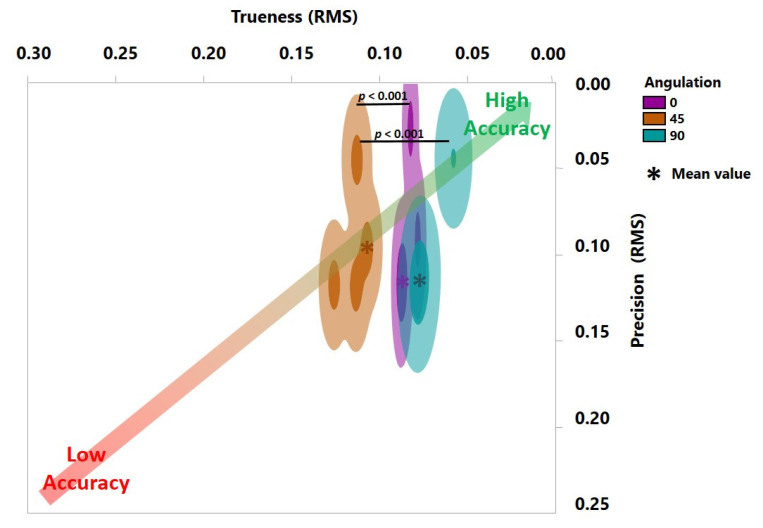
Accuracy of SLA-printed denture bases with three different build angles. Trueness is depicted horizontally and precision vertically. The *p*-value here with regard to trueness was calculated using Tukey’s analysis. No statistically significant difference was detected for precision.

**Figure 5:**
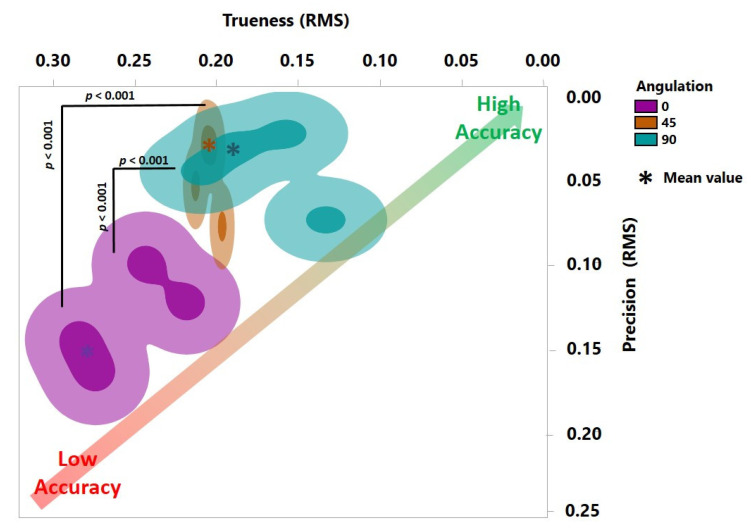
Accuracy of DLP-printed denture bases with three different build angles. Trueness is depicted horizontally and precision vertically. The *p*-value here with regard to trueness and precision was calculated using Tukey’s analysis.

The Shapiro–Wilk test revealed a normal distribution of the gathered data. As shown in Figure 4 and Figure 5, a statistically significant interaction was found in the trueness (F (2, 24) = 10.78, *p* = 0.0005). Additionally, each main effect showed significant differences: AM methods (F (1, 24) = 164.7, *p* < 0.0001) and build angles (F (2, 24) = 16.39, *p* = 0.0744, *p* < 0.0001). Furthermore, the overall tendency indicated that SLA denture bases had significantly higher trueness for most build angles compared to DLP (*p* < 0.001), confirmed by Tukey’s multiple comparison tests.

Regarding the precision, there was a statistically significant interaction (F (2, 18) = 6.044, *p* = 0.0098). Meanwhile, the main effect of build angles had significant differences (F (2, 18) = 4.061, *p* = 0.0350) while AM methods showed no significant differences (F (1, 18) = 1.907, *p* = 0.1842). Specifically, a post hoc multiple comparison test demonstrated significant greater precision in the DLP of 0° (0.134 ± 0.028) in comparison to the DLP of 45° (0.048 ± 0.023, *p* = 0.0151) and 90° (0.044 ± 0.023, *p* = 0.0098).

**Figure 6:**
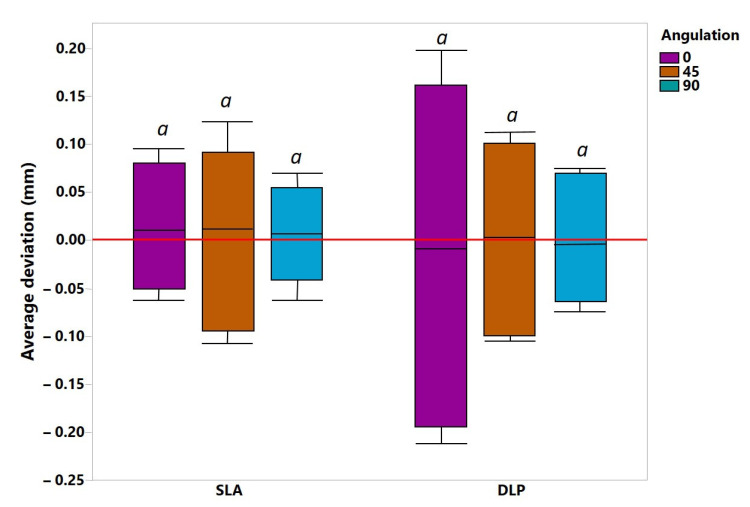
The mean average deviations between various study groups over the whole intaglio surface. *a* represents level of statistical significance.

**Figure 7:**
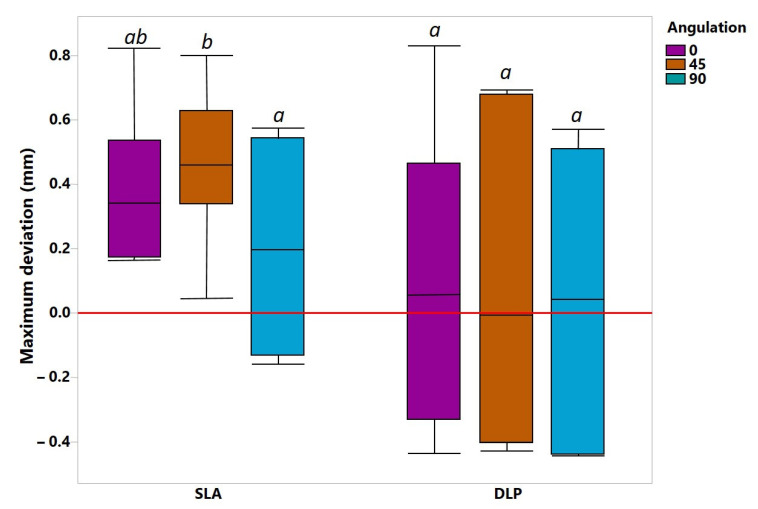
The mean maximum deviations between various study groups over the whole intaglio surface. *a*, *ab*, *b*—represent levels of statistical significance.

As shown in Figure 6, with regard to the mean deviation, no statistically significant differences were observed: interaction (F (2, 54) = 0.06212, *p* = 0.9398), AM methods (F (1, 54) = 0.1555, *p* = 0.6948) and build angles (F (2, 54) = 0.0022, *p* = 0.9978). However, in terms of the maximum deviation, significantly higher inaccuracies up to 0.5 mm were observed for the SLA group in 0° and 45° orientations compared to DLP (Figure 7). AM methods as the main factor showed statistically significant differences in mean maximum deviation (F (1, 54) = 7.053, *p* = 0.0104), confirmed by a two-way ANOVA.

**Figure 8:**
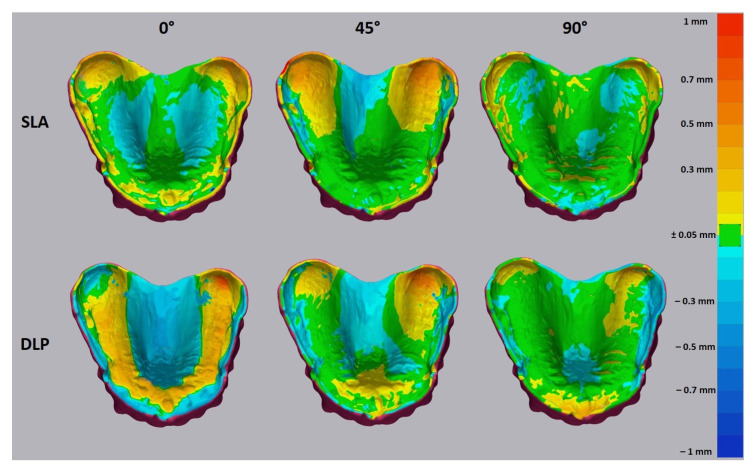
The heat map for the trueness of 3D printed denture bases. The tolerance level was set to ±0.05 mm. The yellow to red deviation shows the positive deviations and cyan to blue, negative.

**Figure 9:**
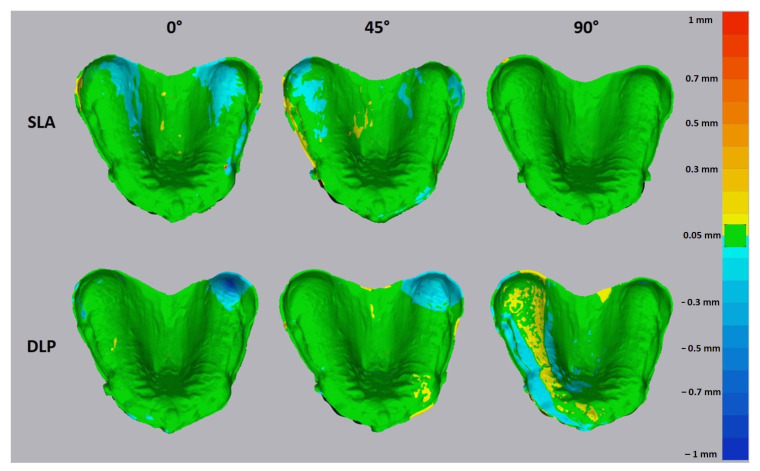
The heat map for the precision of 3D printed denture bases. The tolerance level was set to ±0.05 mm. The yellow to red deviation shows the positive deviations and cyan to blue, negative.

The trueness heat map demonstrated positive deviations (yellow to red) in the area of tuber maxillae and negative deviations (cyan to blue) in the palatal area for SLA bases printed with 0° and 45° orientations (Figure 8 and Figure 9). Only shallow deviations could be observed in the 90° printed SLA bases. The intaglio surface of 0° printed DLP bases showed the poorest accuracy and was almost fully distorted in a positive way on the alveolar residual ridge and in a negative way on the palate and lingual slope. The 90° printed DLP bases showed the most uniform intaglio surface with fewer deviations.

**Figure 10:**
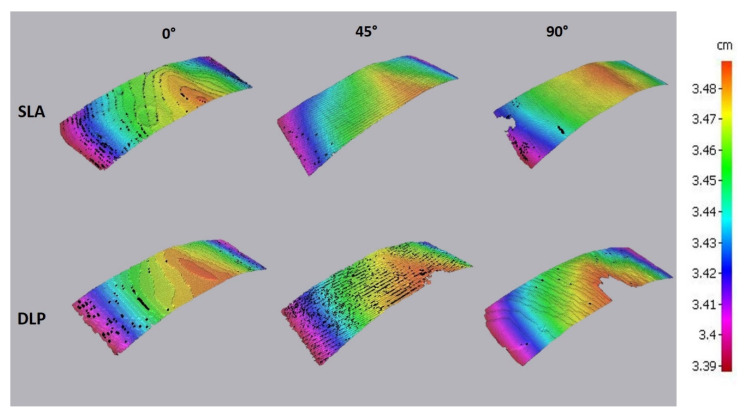
The optical 3D metrology showing differences in layer orientation for SLA and DLP specimens.

The optical 3D metrology test displayed a significant difference in surface structure (Figure 10). A strongly pronounced staircase effect was demonstrated for both SLA and DLP 0° printed specimens. No significant differences were observed for 45° specimens. Despite the same orientation, the 90° specimens showed isotropic surface structures, whereby the SLA base demonstrated the most uniform surface devoid of any staircase effect.

## 4. Discussion

### 4.1. Outcomes of the Accuracy Test

The accuracy analysis in the present study revealed greater trueness for SLA compared to DLP. Furthermore, the 90° orientation exhibited fewer deviations for both SLA and DLP methods. Therefore, both null hypotheses were rejected.

The greater deviations of the intaglio surface in the 0° and 45° groups may be attributed to the more pronounced staircase effect, which is related to the layer-wise building process. This exerted negative effects on the trueness of the palatal surface, including grooves and line angles [26]. In general, the large curved surfaces are more prone to the staircase effect than vertical surfaces, which leads to higher dimensional errors [27,28].

The optical 3D metrology analysis revealed that the SLA-printed bases demonstrated less of a staircase effect than DLP in all orientation groups. This fact may be attributed to the inherent process-related difference between these two vat polymerization methods. Thus, even if the layers are oriented perpendicular to the intaglio surface, the staircase effect may be caused by the light projection of the square-shaped 2D pixel patterns through the mirror device, which generates each voxel [29].

The majority of accuracy-related studies have been carried out using the DLP method. You et al. investigated the accuracy of an SLA-printed trial denture in beige material and reported the RMS values for the intaglio surface with 50 µm layer thickness in the order of 0.152 ± 0.01, which is in agreement with the results of the present study [26]. However, the heat map of You et al. revealed much higher centripetal shrinkage, as observed for SLA bases in the present case. An objective comparison between these two studies with regard to the localization of the deviations is restricted by the unclear build angle and utilization of the trial denture beige material in You et al.

Hada et al. investigated the accuracy of SLA-printed dentures using transparent material (Clear, Formlabs, Somerville, MA, USA) and a Form 2 printer (Formlabs, Somerville, USA) with 100 µm layer thickness and reported the 45° orientation to provide the superior trueness [24]. It must be stressed that this material is not verified by the manufacturer for denture base fabrication nor for dental applications. For this reason, in the present study, the Denture Base OP Resin (Formlabs, Somerville, MA, USA) and Form 3B printer (Formlabs, Somerville, MA, USA) were utilized for comparative analysis with V-Print dentbase (VOCO GmbH, Cuxhaven, Germany) and the Solflex 350 Plus (W2P Engineering GmbH, Vienna, Austria) DLP method. Contrary to Hada et al., the outcomes of the present study propose the 90° angle to be an optimal build angle for denture base fabrication. Rubayo et al. investigated the accuracy of SLA-produced surgical guides using 100 µm layer thickness and revealed the 45° angle to perform best in terms of geometrical accuracy [22]. The contradiction between these studies and the present one may be attributed to an alternative layer thickness of 50 µm or the different materials, as used in this study. This assumption is further supported by the outcomes of Dalal et al., who postulated that 50 µm may be more accurate than 100 µm for the SLA printing process [23].

### 4.2. Clinical Interpretation

The observed discrepancies in RMS value between SLA and DLP methods need careful clinical interpretation. For this reason, the additional analysis of the mean deviation in mm was performed. This has shown that the mean deviation for SLA and DLP bases did not exceed a value of ±0.02 mm. This is contradictory with the study of Hwang et al., as they reported values of ±0.06 mm for DLP, and Yoon et al. reported values up to 0.5 mm. [11,13]. This might be due to the layer thickness of 0.1 mm, build angle of 100° and an alternative material used in both studies.

The mean maximum deviation reached a value of 0.5 mm for 0° and 45° SLA bases, which is significantly higher as compared to DLP (up to 0.1 mm). Furthermore, the SLA bases predominantly showed deviation of a positive manner in contrast to DLP.

The majority of negative deviations were found on the palatal area, which may compromise the posterior palatal seal. Further positive deviations were revealed in the stress-bearing areas such as residual alveolar ridge and tuber maxillae, which might cause compression and incongruence in these areas. According to the heat map, the 90° orientation may provide more accurate denture bases for both DLP and SLA, leading to a better tissue adaptation. This disagrees with the study of Jin et al., as they reported the 45° (135°) angle to be an optimal printing angle for DLP according to the heat map [12]. Here, it could be speculated that the different DLP devices used might generate different printing results. Furthermore, another factor might be the postprocessing method used. Studies have shown the influence on the mechanical properties and this could possibly also have an influence on the accuracy of the denture bases (development of negative residual stresses inside the printed parts) [30,31,32].

The present study concentrated on the intaglio surface, which does not necessarily reflect the general accuracy of the whole denture base. Thus, the studies of You et al. showed a certain discrepancy in the accuracy of intaglio, cameo and socketed surfaces for both SLA and DLP [26,33]. The manufacturing accuracy of the denture socketed area using SLA should be considered in future studies.

It must be emphasized that in the majority of the studies devoted to the accuracy of DLP-printed denture bases, a dental NextDent 3D printer was used with a layer thickness of 100 µm and a wavelength of 405 nm [7,8,12,26,34]. The printing hard- and software may vary depending on the manufacturer. So, there are more active factors than just the illumination source (SLA, DLP, liquid crystal display (LCD)). The DLP printer used in this study had a moving DLP projector with 385 nm, which increased the maximum usable area, but might lead to additional inaccuracy. In addition, different vat systems are now available on the market, which could also have an impact on precision, as pull-off forces, reduced light intensity due to “clouding” and unevenness in the vat bottom may also affect the result. A limitation of this study is also that multiple identical objects had to be printed in the same position, which can result in increased wear of the tray bottom, which can be reflected in the precision and accuracy. Further studies on the topic should include all available hardware options for DLP and SLA 3D printing of denture bases.

The part geometry also has a considerable influence on the accuracy, depending on the part alignment. The formation of suction cups during part generation can lead to considerable pull-off forces, which can result in subsequent distortion of the part. The prosthesis bases used in this study showed suction cup formation, especially in the 90° orientation. Therefore, accuracy may presumably depend on both component orientation and component geometry.

Additionally, the material itself is known to influence the geometrical accuracy [34]. The abovementioned facts cater for further research considering a comparison of various 3D printers within the one group of illumination technologies, utilization of diverse geometries of build parts and utilization of verified materials.

## 5. Conclusions

Within the limitations of the present in vitro study, the following conclusions were drawn:

- SLA may produce an intaglio denture surface with a better trueness than DLP;
- SLA and DLP demonstrated nearly the same precision for 3D printing of denture bases;
- The build orientation of 90° may provide the best trueness for both SLA and DLP;
- Besides the illumination source of a 3D printing hardware (SLA, DLP, LCD), the geometrical accuracy may be presumably influenced by part geometry and material type.

## Figures and Tables

**Table 1:** The root mean square (RMS) values for trueness, precision and the average and maximum deviations in mm between various study groups.

		SLA (Stereolithography)			DLP (Direct Light Processing)		
		0°	45°	90°	0°	45°	90°
Trueness (RMS)	Mean	0.094	0.132	0.083	0.256	0.211	0.163
	SD	0.004	0.016	0.009	0.031	0.031	0.030
Precision (RMS)	Mean	0.087	0.094	0.098	0.134	0.048	0.044
	SD	0.042	0.034	0.037	0.028	0.023	0.023
Average + (mm)	Mean	0.082	0.099	0.055	0.166	0.101	0.066
	SD	0.011	0.015	0.009	0.027	0.010	0.010
Average − (mm)	Mean	−0.054	−0.089	−0.045	−0.187	−0.097	−0.065
	SD	0.006	0.018	0.010	0.024	0.008	0.006
Max + (mm)	Mean	0.613	0.630	0.533	0.547	0.573	0.500
	SD	0.136	0.119	0.030	0.169	0.163	0.071
Max − (mm)	Mean	−0.168	−0.294	−0.132	−0.366	−0.402	−0.416
	SD	0.005	0.143	0.020	0.058	0.027	0.048

## Data Availability

All study data is available on request.

## References

[1] Wemken G., Spies B.C., Pieralli S., Adali U., Beuer F., Wesemann C. (2020). Do hydrothermal aging and microwave sterilization affect the trueness of milled, additive manufactured and injection molded denture bases?. J. Mech. Behav. Biomed. Mater..

[2] Schweiger J., Stumbaum J., Edelhoff D., Güth J.-F. (2018). Systematics and concepts for the digital production of complete dentures: Risks and opportunities. Int. J. Comput. Dent..

[3] Schweiger J., Güth J.-F., Edelhoff D., Stumbaum J. (2017). Virtual evaluation for CAD-CAM-fabricated complete dentures. J. Prosthet. Dent..

[4] Lin W.-S., Harris B.T., Pellerito J., Morton D. (2018). Fabrication of an interim complete removable dental prosthesis with an in-office digital light processing three-dimensional printer: A proof-of-concept technique. J. Prosthet. Dent..

[5] Unkovskiy A., Wahl E., Zander A.T., Huettig F., Spintzyk S. (2019). Intraoral scanning to fabricate complete dentures with functional borders: A proof-of-concept case report. BMC Oral Health.

[6] Bilgin M.S., Erdem A., Aglarci O.S., Dilber E. (2015). Fabricating Complete Dentures with CAD/CAM and RP Technologies. J. Prosthodont..

[7] Cristache C.M., Totu E.E., Iorgulescu G., Pantazi A., Dorobantu D., Nechifor A.C., Isildak I., Burlibasa M., Nechifor G., Enachescu M. (2020). Eighteen Months Follow-Up with Patient-Centered Outcomes Assessment of Complete Dentures Manufactured Using a Hybrid Nanocomposite and Additive CAD/CAM Protocol. J. Clin. Med..

[8] Inokoshi M., Kanazawa M., Minakuchi S. (2012). Evaluation of a complete denture trial method applying rapid prototyping. Dent. Mater. J..

[9] Berman B. (2012). 3-D printing: The new industial revolution. Bus. Horiz..

[10] Yoon H.-I., Hwang H.-J., Ohkubo C., Han J.-S., Park E.-J. (2018). Evaluation of the trueness and tissue surface adaptation of CAD-CAM mandibular denture bases manufactured using digital light processing. J. Prosthet. Dent..

[11] Hwang H.-J., Lee S.J., Park E.-J., Yoon H.-I. (2019). Assessment of the trueness and tissue surface adaptation of CAD-CAM maxillary denture bases manufactured using digital light processing. J. Prosthet. Dent..

[12] Jin M.-C., Yoon H.-I., Yeo I.-S., Kim S.-H., Han J.-S. (2020). The effect of build angle on the tissue surface adaptation of maxillary and mandibular complete denture bases manufactured by digital light processing. J. Prosthet. Dent..

[13] Yoon S.-N., Oh K.C., Lee S.J., Han J.-S., Yoon H.-I. (2020). Tissue surface adaptation of CAD-CAM maxillary and mandibular complete denture bases manufactured by digital light processing: A clinical study. J. Prosthet. Dent..

[14] Stansbury J.W., Idacavage M.J. (2016). 3D printing with polymers: Challenges among expanding options and opportunities. Dent. Mater..

[15] Gibson I., Rosen D.W., Stucker B. (2015). Additive Manufacturing Technologies.

[16] Taormina G., Sciancalepore C., Messori M., Bondioli F. (2018). 3D printing processes for photocurable polymeric materials: Technologies, materials, and future trends. J. Appl. Biomater. Funct. Mater..

[17] Unkovskiy A., Roehler A., Huettig F., Geis-Gerstorfer J., Brom J., Keutel C., Spintzyk S. (2019). Simplifying the digital workflow of facial prostheses manufacturing using a three-dimensional (3D) database: Setup, development, and aspects of virtual data validation for reproduction. J. Prosthodont. Res..

[18] Unkovskiy A., Bui P.H.-B., Schille C., Geis-Gerstorfer J., Huettig F., Spintzyk S. (2018). Objects build orientation, positioning, and curing influence dimensional accuracy and flexural properties of stereolithographically printed resin. Dent. Mater..

[19] Ollison T., Berisso K. (2010). Three-dimensional printing build variables that impact cylindricity. J. Ind. Technol..

[20] Aretxabaleta M., Xepapadeas A.B., Poets C.F., Koos B., Spintzyk S. (2021). Comparison of additive and subtractive CAD/CAM materials for their potential use as Tübingen Palatal Plate: An in-vitro study on flexural strength. Addit. Manuf..

[21] Choi J.-W., Ahn J.-J., Son K., Huh J.-B. (2019). Three-Dimensional Evaluation on Accuracy of Conventional and Milled Gypsum Models and 3D Printed Photopolymer Models. Materials.

[22] Rubayo D.D., Phasuk K., Vickery J.M., Morton D., Lin W.-S. (2020). Influences of build angle on the accuracy, printing time, and material consumption of additively manufactured surgical templates. J. Prosthet. Dent..

[23] Dalal N., Ammoun R., Abdulmajeed A.A., Deeb G.R., Bencharit S. (2020). Intaglio Surface Dimension and Guide Tube Deviations of Implant Surgical Guides Influenced by Printing Layer Thickness and Angulation Setting. J. Prosthodont..

[24] Hada T., Kanazawa M., Iwaki M., Arakida T., Soeda Y., Katheng A., Otake R., Minakuchi S. (2020). Effect of Printing Direction on the Accuracy of 3D-Printed Dentures Using Stereolithography Technology. Materials.

[25] You S.-G., Kang S.-Y., Bae S.-Y., Kim J.-H. (2020). Evaluation of the adaptation of complete denture metal bases fabricated with dental CAD-CAM systems: An in vitro study. J. Prosthet. Dent..

[26] You S.-M., Kang S.-Y., Bae S.-Y., Kim J.-H. (2021). Evaluation of the accuracy (trueness and precision) of a maxillary trial denture according to the layer thickness: An in vitro study. J. Prosthet. Dent..

[27] Patzelt S.B., Bishti S., Stampf S., Att W. (2014). Accuracy of computer-aided design/computer-aided manufacturing–generated dental casts based on intraoral scanner data. J. Am. Dent. Assoc..

[28] Choi S., Chan A. (2004). A virtual prototyping system for rapid product development. Comput. Des..

[29] Formlabs SLA vs. DLP: Guide to Resin 3D Printers. https://formlabs.com/blog/resin-3d-printer-comparison-sla-vs-dlp/.

[30] Xu Y., Xepapadeas A.B., Koos B., Geis-Gerstorfer J., Li P., Spintzyk S. (2021). Effect of post-rinsing time on the mechanical strength and cytotoxicity of a 3D printed orthodontic splint material. Dent. Mater..

[31] Reymus M., Stawarczyk B. (2020). Influence of Different Postpolymerization Strategies and Artificial Aging on Hardness of 3D-Printed Resin Materials: An In Vitro Study. Int. J. Prosthodont..

[32] Reymus M., Lümkemann N., Stawarczyk B. (2019). 3D-printed material for temporary restorations: Impact of print layer thickness and post-curing method on degree of conversion. Int. J. Comput. Dent..

[33] You S.-M., Lee B.-I., Kim J.-H. (2020). Evaluation of trueness in a denture base fabricated by using CAD-CAM systems and adaptation to the socketed surface of denture base: An in vitro study. J. Prosthet. Dent..

[34] Kulkarni P., Marsan A., Dutta D. (2000). A review of process planning techniques in layered manufacturing. Rapid Prototyp. J..

